# Toxicity Assessments of Chalcone and Some Synthetic Chalcone Analogues in a Zebrafish Model

**DOI:** 10.3390/molecules19010641

**Published:** 2014-01-07

**Authors:** Ya-Ting Lee, Tsorng-Harn Fong, Hui-Min Chen, Chao-Yuan Chang, Yun-Hsin Wang, Ching-Yuh Chern, Yau-Hung Chen

**Affiliations:** 1 Department of Chemistry, Tamkang University, 151, Yingzhuan Road, Danshui Dist., New Taipei City 25137, Taiwan; E-Mails: joanna20520@gmail.com (Y.-T.L.); frank00634@hotmail.com (C.-Y.C.); 129180@mail.tku.edu.tw (Y.-H.W.); 2 Department of Anatomy, School of Medicine, College of Medicine, Taipei Medical University, Taipei 11031, Taiwan; E-Mails: thfong@tmu.edu.tw (T.-H.F.); chm7805@tmu.edu.tw (H.-M.C.); 3 Department of Applied Chemistry, National Chia-Yi University, Chia-Yi 60004, Taiwan

**Keywords:** chalcone, embryogenesis, muscle, toxicity, zebrafish

## Abstract

The aim of this study was to investigate the *in vivo* toxicities of some novel synthetic chalcones. Chalcone and four chalcone analogues **1a**–**d** were evaluated using zebrafish embryos following antibody staining to visualize their morphological changes and muscle fiber alignment. Results showed that embryos treated with 3'-hydroxychalcone (compound **1b**) displayed a high percentage of muscle defects (96.6%), especially myofibril misalignment. Ultrastructural analysis revealed that compound **1b**-treated embryos displayed many muscle defect phenotypes, including breakage and collapse of myofibrils, reduced cell numbers, and disorganized thick (myosin) and thin (actin) filaments. Taken together, our results provide *in vivo* evidence of the myotoxic effects of the synthesized chalcone analogues on developing zebrafish embryos.

## 1. Introduction

Chalcone (1,3-diphenyl-2-propen-1-one), is an important compound for the biosynthesis of flavonoids. Chalcones have a general structure consisting of two phenyl groups, both with hydroxyl group, connected by a C3 bridge. Natural occurring chalcones, as well as synthetic chalcone analogues, have been demonstrated to possess many pharmaceutical effects including anti-inflammatory, anti-oxidant, anti-nociceptive, anti-parasites, and anti-proliferative activities [1,2,3,4,5,6,7]. Moreover, some chalcones can be conjugated or hybridized with other compounds and thus become potential anti-cancer therapeutic agents [8,9,10]. These observations highlight the importance and multiple applications of chalcones. However, current knowledge regarding the toxic effects of chalcones in vertebrates during embryogenesis is still limited.

To date, many kinds of chalcones have been reported, including synthetic and natural ones. For their toxicity assessment, most previous studies used cancer cell lines [11,12,13], some used rat liver epithelial cells [14], and only few reports used rat (or dog) as a model to test the embryotoxicity and teratogenicity of some specific chalcones [15]. To exand our knowledge on chalcone toxicity (especially embryotoxicity), development of an alternative model is essential. The optical transparency and clear developmental stages of zebrafish (*Danio rerio*) embryos allows noninvasive and dynamic evaluation of embryotoxicity *in vivo.* In this study, we used embryonic zebrafish as a model to assess the toxic effects of chalcones (chalcone and some chalcone analogues) on muscle development *in vivo*. We generated a series of time- and dose-dependent chalcone exposure experiments. Subtle changes in the muscle fiber alignment can be easily observed by staining with specific monoclonal antibodies. This strategy is excellent for studying chalcone-induced myotoxicity during early embryonic development.

## 2. Results and Discussion

### 2.1. Chemistry

**Figure 1:**
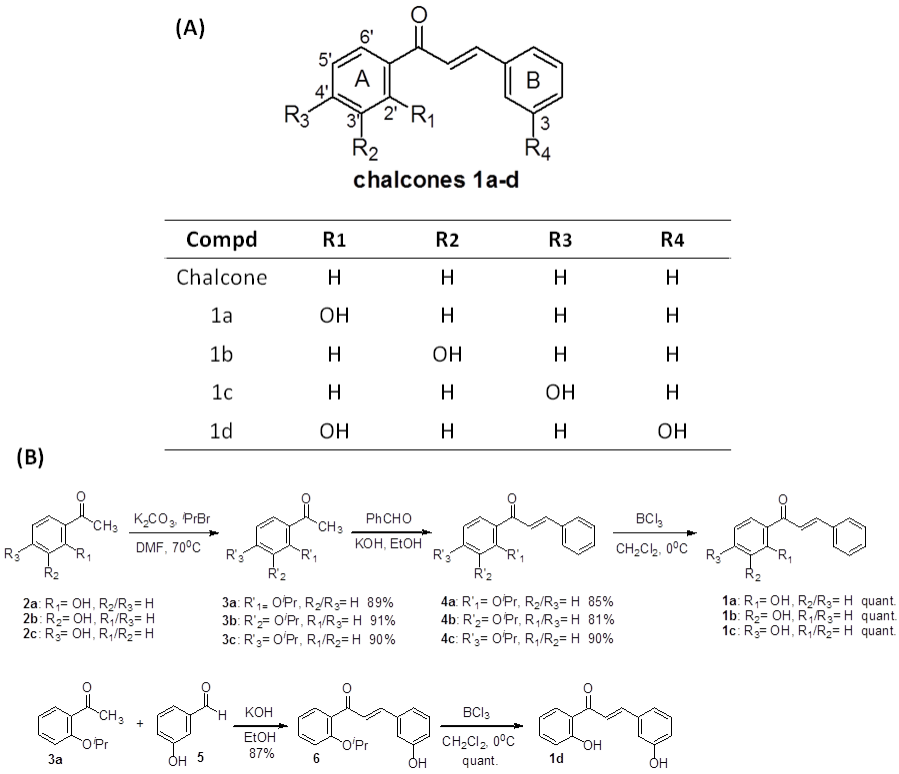
(**A**) Structures of chalcone and compounds **1a**–**d**; (**B**) Synthesis of compounds **1a**–**d**.

For this study, we synthesized four chalcones **1a**–**d** (Figure 1A). These aldol compounds were obtained using a procedure similar to one described previously [16,17,18,19]. Unfortunately, the reaction yields using this method was very low for the chalcones **1a** and **1d** (~15%). It is possible that intramolecular hydrogen bonding such as those observed in **1a** or **1d** prevents the aldol reaction. Therefore, *O*-isoproxylacetophenones **3a**, **3b**, and **3c** were used as the starting material for our synthesis. The preparations of *O*-isoproxylacetophenones **3a**–**c** were straightforward. Acetophenones **2a**–**c** were protected with isopropyl bromide and potassium carbonate in DMF in excellent yield. (89%, 91% and 90%, respectively). The isolated products **3a**–**c** were then reacted with appropriate benzaldehydes and 5 N KOH to provide intermediates **4a**–**c** and **6** in 85%, 81%, 90% and 87% yields. The *O*-isopropyl ether group was removed quantitatively with BCl_3_ to afford the target chalcones **1a**, **1b**, **1c** and **1d** (Figure 1B).

### 2.2. Titration and Survival Rates Analysis

**Figure 2:**
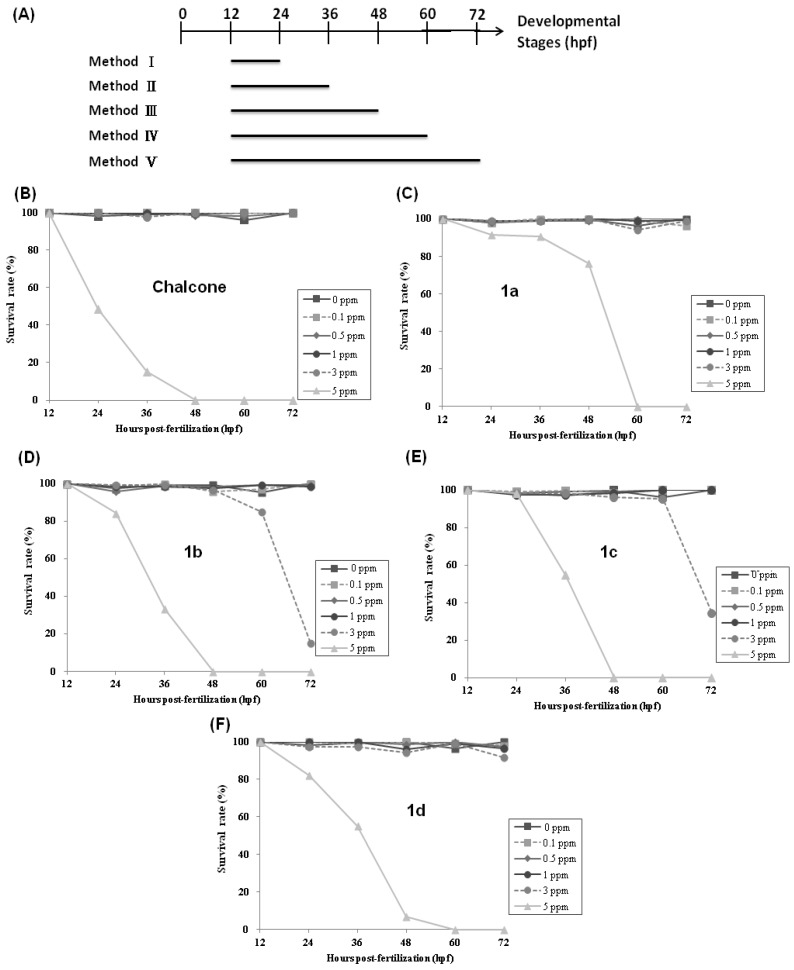
(**A**) Exposure protocols used in this study; (**B**–**E**) Survival analysis of zebrafish embryos after chalcone, compounds **1a**–**d** treatment.

In order to access the toxic effects of chalcone and the synthetic chalcone analogues **1a**–**d** on zebrafish larvae, first of all, we treated zebrafish embryos with low dosages of compounds (0.1, 0.5 and 1 ppm) via exposure methods I–V (12–24, 12–36, 12–48, 12–60, 12–72 hpf; Figure 2A) to calculate the survival rates. As shown in Figure 2B–E, 99.2%–100% of the no treatment control (mock, 0 ppm) embryos and 96.4%–100% of chalcone and compounds **1a**–**d**-treated embryos were alive after exposure via exposure protocols I–V. No significant differences in the survival rates were observed between no treatment control and low-dosage-treated groups (0.1, 0.5 and 1 ppm). Next, we treated zebrafish embryos with high dosages of chalcone and compounds **1a**–**d** (3 and 5 ppm) via exposure methods I–V to calculate the survival rates. Results showed that the survival rates decreased as the time of exposure and the concentration (3 and 5 ppm) of chalcone and compounds **1a**–**d** increased. At the end of the examination (72 h postfertilization, hpf), almost no embryos survived treatment with 5 ppm of chalcone and compounds **1a**–**d**. On the other hand, we noticed that 3 ppm of compound **1b**-exposed embryos (via method II: 12–36 hpf) displayed high survival rates (99.3%) and high percentage of malformed phenotypes (96.6%). Therefore, we used compound **1b**-exposed embryos via exposure method II (12–36 hpf) as material for subsequent analysis.

### 2.3. Phenotypic Changes after Chalcone Treatment

**Figure 3:**
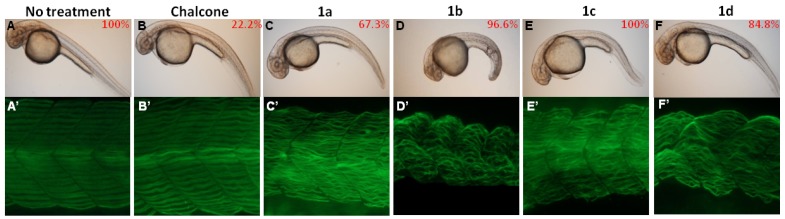
Chalcones exposure affects myofibril alignment. (**A**–**F**) Visible and defective phenotypes of zebrafish embryos after chalcones treatment. Embryos were exposed to water (no treatment control; A) or water containing 3 ppm of chalcones (including chalcone and compounds **1a**–**d**; (**A**’–**F**’) F59 monoclonal antibody staining of zebrafish embryo derived from each groups. All the photos were taken from the lateral view and were of developmental stages at 36 hpf. The exposure duration ranges from 12 to 36 hpf.

To explore the toxicities of the chalcones (chalcone and the synthetic chalcone analogues **1a**–**d**) during zebrafish embryogenesis, we exposed zebrafish embryos to different chalcones (3 ppm) via exposure methods II (12–36 hpf), and recorded their malformation phenotypes. Results showed that embryos derived from the compounds **1a**–**d** (3 ppm)-treated groups displayed shorter body length, curved trunks and malformed somite boundary than those with either no treatment control or the chalcone-treated group (Figure 3A *vs.* 3B–F). For further investigation, the monoclonal antibody F59 was employed to visualize the alignments of muscle fibers in no treatment control and chalcones (including chalcone and compounds **1a**–**d**)-treated zebrafish embryos. In both no treatment (mock) control and chalcone-treated embryos, muscle fibers aligned well in the chevron-shaped somatic hemi-segment (Figure 3A’,B’). In compounds **1a**–**d**-treated embryos, muscle fibers were both split and short by 36 hpf, and lost their integrity and aligned disorderly (Figure 3C’–F’). These observations suggested that synthetic chalcone analogues (compounds **1a**–**d**) are myotoxic and can impair myofibril alignment.

### 2.4. Chalcones Affect Myofibril Ultrastructures and Alignment

**Figure 4:**
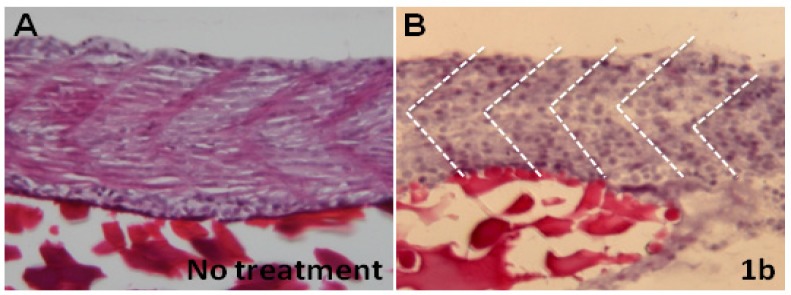
Compound **1b** affects myofibril alignment. Representative images of hemetoxylin (H) & eosin Y (E) staining of zebrafish embryo after exposure to water (**A**) or water containing 3 ppm of compound **1b**. (**B**) Dashed lines mark the boundary of somites.

**Figure 5:**
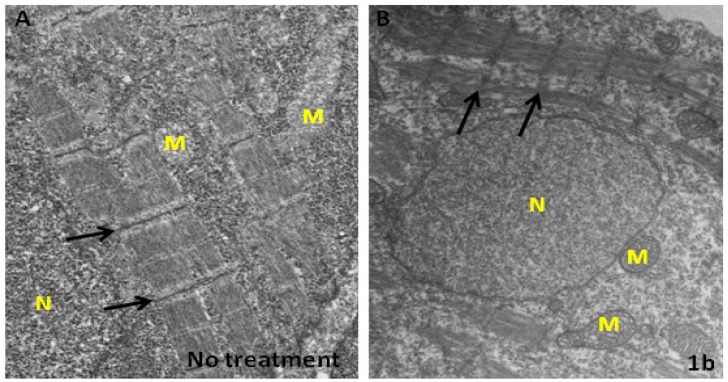
Compound **1b** affects ultrastructure of myofibril. Representative images of TEM of zebrafish embryo after exposure to water (**A**) or water containing 3 ppm of compound **1b**. (**B**) Arrows indicate the positions of Z-line. M: mitochondria; N: nucleus.

We have shown that chalcone-treated embryos have a shorter body length and curved trunk. Is it possible such malformations are due to the disorganization of myofibrils? To address this question, we carried out hematoxylin and eosin Y (H&E) staining and electron microscopy (EM) experiments. After H&E staining, boundaries of the segments of myofibrils in the embryos derived from the no treatment control group are compact with normal morphology (Figure 4A). In contrast, myofibrils’ organization are fractured in the compound **1b**-treated embryos (Figure 4B). Ultrastructural analysis indicated that in compound **1b**-treated embryos many myofibrils are broken and collapsed. The numbers of myofibrils is greatly decreased. The remainder are thinner and with fragmentary cytosolic components. Although the Z-line is still visible, the thick (myosin) and thin (actin) filaments are disorganized (Figure 5A *vs*. 5B). Notably, cytosolic electron density in the compound **1b**-treated embryos is more greatly decreased than in the no treatment control group (Figure 5B *vs*. 5A). However, mitochondria electron density in the compound **1b**-treated embryos is increased, indicating that some substances are being produced or accumulated in the mitochondria. Taken together, we concluded that compound **1b** affects myofibril alignment and organization.

## 3. Experimental

### 3.1. General Information

Proton (300 MHz) and carbon (75 MHz) NMR spectra were recorded on a Varian Mercury-300 NMR spectrometer (Agilent, Santa Clara, CA, USA). Cchemical shifts are reported on the δ scale as parts per million (ppm) downfield from tetramethylsilane (TMS) used as internal reference. Mass spectra were measured with a VG Analytical Model 70–250 s Mass Spectrometer (Varian, Palo Alto, CA, USA). All reagents were used as obtained commercially.

### 3.2. Synthesis of Chalcone Analogues

A mixture of the corresponding acetophenone **2a**–**c** (1 equiv), isopropyl bromide (1.1 equiv), and potassium carbonate (1.1 equiv) in DMF (0.3 M) was stirred at 70 °C for 3 h. Then, the reaction mixture was added to water and extracted with CH_2_Cl_2_. The purified *O*-isopropyl product **3a**–**c** was obtained in excellent yield by column chromatography (silicagel, 70–230 mesh) using CH_2_Cl_2_ as eluent. The isolated product **3a**–**c** (1 equiv) was then reacted with the appropriate benzaldehyde (1 equiv) and 5N KOH at room temperature until the aldehyde was consumed. After that, HCl (10%) was added until neutrality. In the cases where the chalcones precipitated, they were filtered and crystallized from MeOH. In the other cases, the product was purified using column chromatography with EtOAc/hexane as eluent. The *O*-isopropyl ether groups of **4a**–**c**, **6** (1 equiv) were removed quantitatively with BCl_3_ (1.1 equiv) in CH_2_Cl_2_ (0.3 M) at 0 °C for 2 h to afford the target chalcones **1a**–**d**.

*1-(2-Hydroxyphenyl)-3-phenylpropenone* (**1a**). Mp 89–90 (lit. 89 °C) [20]; ^1^H-NMR (CDCl_3_): 12.80 (1H, s, -OH), 7.93 (1H, d, *J* = 15.6 Hz), 7.93 (1H, d, *J* = 8.0 Hz), 7.66 (1H, d, *J* = 15.6 Hz), 7.68–7.65 (1H, m), 7.55–7.42 (5H, m), 7.03 (1H, dd, *J* = 8.0 Hz, 1.0 Hz), 6.95 (1H, td, *J* = 7.2 Hz, 1.0 Hz); ^13^C-NMR (CDCl_3_): 193.8, 163.5, 145.4, 136.4, 134.5, 130.9, 129.6, 129.0, 128.6, 120.0, 119.9, 118.8, 118.6; EI-MS *m/z* (rel.int.%): 224 [M^+^,15], 175 (18), 131 (18), 119 (100), 91 (94), 77 (28), 65 (33).

*1-(3-Hydroxyphenyl)-3-phenylpropenone* (**1b**). Mp 120–121 °C (lit. 120.2–120.6 °C) [17]; ^1^H-NMR (CDCl_3_): 7.83 (1H, d, *J* = 15.6 Hz), 7.66–7.58 (3H, m), 7.51 (1H, d, *J* = 15.6 Hz), 7.45–7.34 (5H, m), 7.14 (1H, ddd, *J* = 8.0 Hz, 2.4 Hz, 1.2 Hz), 4.8–4.2 (1H, br s, -OH); ^13^C-NMR (CDCl_3_):190.9, 156.5, 145.5, 139.4, 134.7, 130.7, 129.9, 129.0, 128.6, 121.9, 120.9, 120.5, 115.2; EI-MS *m/z* (rel.int.%): 224 [M^+^,100], 223 (79), 195 (14), 121 (40), 103 (20), 93 (19), 65 (19).

*1-(4-Hydroxyphenyl)-3-phenylpropenone* (**1c**). Mp 179–180 °C (lit. 175–179 °C) [19]; ^1^H-NMR (CDCl_3_): 8.00 (2H, d, *J* = 8.8 Hz), 7.80 (1H, d, *J* = 15.6 Hz), 7.63 (2H, dd, *J* = 2.6 Hz, 2.0 Hz), 7.53 (1H, d, *J* = 15.6 Hz), 7.42–7.40 (3H, m), 6.93 (2H, d, *J* = 8.8 Hz), 2.10–1.90 (1H, br s, -OH); ^13^C-NMR (CDCl_3_):188.4, 162.9, 143.8, 136.4, 132.0, 131.2, 131.0, 129.8, 129.4, 123.0, 116.2; EI-MS *m/z* (rel.int.%): 224 [M^+^,100], 223 (79), 103 (15), 65 (20).

*1-(2-Hydroxyphenyl)-3-(3-hydroxyphenyl)propenone* (**1d**). Mp 180–181 °C (lit. 180–181 °C) [21]; ^1^H-NMR (acetone-*d*_6_): 12.87 (1H, br s, -OH), 8.60 (1H, br s, -OH), 8.26 (1H, dd, *J* = 8.4 Hz, 1.6 Hz), 7.98 (1H, d, *J* = 15.6 Hz), 7.85 (1H, d, *J* = 15.6 Hz), 7.56 (1H, ddd, *J* = 8.6 Hz, 8.6 Hz, 1.6 Hz), 7.38–7.25 (3H, m), 7.03–6.94 (3H, m); ^13^C-NMR (acetone-*d*_6_):195.0, 164.5, 158.7, 146.3, 137.4, 137.1, 131.3, 130.9, 121.4, 121.3, 120.8, 119.8, 119.0, 118.9, 116.2; EI-MS *m/z* (rel.int.%): 240 [M^+^,100], 239 (79), 191 (15), 147 (18), 107 (81), 65 (33).

### 3.3. Fish Care and Chemicals Treatment

Mature zebrafish (AB strain) was supplied by the Zebrafish Core at Academia Sinica (ZCAS, Taipei, Taiwan). Embryos were produced using standard procedures [22,23,24] and were staged according to standard criteria: hours postfertilization, hpf; or days postfertilization (dpf) [25].

### 3.4. Histology, Antibody Labeling and Images

The procedures for H&E staining, antibody labeling and cryosection have been described previously [26,27,28,29], except that F59 (Hybridoma Bank, Iowa City, IA, USA) was used as primary antibody. The procedures of embedding and cryosectioning described by Chen and Tsai [30] were followed, except that embryos developed at 36 hpf were used and sections of 10 μm were obtained. All embryos were observed under a microscope (DM 2500, Leica, Wetzlar, Germany) equipped with Nomarski differential interference contrast optics and a fluorescent module having GFP and DsRed filter cubes (Leica). Images of embryos were captured at specific stages with a digital carema (Sony, Tokyo, Japan), or were examined by a Leica SP2 confocal microscope.

### 3.5. Electron Microscopy

The zebrafish embryos after exposure to water or water containing compound **1b** were fixed with 2% paraformaldehyde and 2.5% glutaraldehyde in 0.1 M cacodylate buffer (pH 7.2) overnight at 4 °C. Subsequently, the embryos were washed in buffer twice for 15 min each and then postfixed using 1% osmium tetroxide in 0.1 M cacodylate buffer for 1 h at room temperature. Samples were dehydrated in an ethanol series and embedded in resin using standard procedures. Ultrathin sections were cut and double-stained with uranyl acetate and lead citrate, and then examined in a Hitachi H-600 electron microscope (Hitachi, Tokyo, Japan).

## 4. Conclusions

This study provide a myotoxic assessment of chalcone and some synthetic chalcone analogues in a zebrafish model. This toxicity information should prove useful for further structure-activity relationship analysis.
